# A peculiar case report of primary iTTP in a previously healthy Saudi man

**DOI:** 10.25122/jml-2024-0415

**Published:** 2025-06

**Authors:** Aishwarya Balasubramanian, Bader AlMurad, Nada AlFarrah, Bakr AbuSamrah, Nawal AbulKhoudoud

**Affiliations:** 1 Coimbatore Medical College, Coimbatore, Tamil Nadu, India; 2 Batterjee Medical College, Jeddah, Saudi Arabia; 3 Department of General Internal Medicine, International Medical Center Hospital, Jeddah, Saudi Arabia

**Keywords:** case report, thrombocytopenia, primary immune-mediated thrombotic thrombocytopenic purpura, plasmapheresis

## Abstract

A 43-year-old Saudi man with prediabetes presented with epigastric pain and thrombocytopenia, initially treated as immune thrombocytopenia with dexamethasone. The patient’s condition worsened as he developed a rash and hematuria, prompting hospital transfer and a severe drop in platelet count. Despite platelet transfusions and steroids, he showed no improvement, leading to a suspected diagnosis of thrombotic thrombocytopenic purpura (TTP). The patient's anti-nuclear antibody (ANA) test result was positive. This may be because individuals are more likely to develop an autoimmune disease for up to 12 years following an acute TTP episode. After initial plasmapheresis sessions, he was admitted to the ICU for further management. Diagnostic workup, including ADAMTS13 assay, confirmed primary immune-mediated thrombotic thrombocytopenic purpura (iTTP), leading to the initiation of plasmapheresis and rituximab. After he responded to therapy, plasma exchange (PEX) was discontinued, and his platelet count began to decline. On the third day after discontinuation, his platelet count decreased to 80,000 x 10^9^/L, necessitating the restart of PEX. During treatment, the patient experienced transient neurological symptoms and developed a pulmonary embolism, which was managed with anticoagulation. Plasmapheresis and immunosuppressive therapy resulted in clinical improvement in stabilizing platelet counts, and he was discharged in good condition after 16 sessions of plasmapheresis and three doses of rituximab. This case highlights the diagnostic challenges in atypical TTP presentations and underscores the importance of promptly identifying TTP and initiating aggressive therapy.

## INTRODUCTION

Thrombotic thrombocytopenic purpura (TTP) is a potentially fatal microangiopathy typically characterized by end-organ ischemia associated with widespread microvascular platelet-rich thrombi, thrombocytopenia, and microangiopathic hemolytic anemia (MAHA) [1]. Non-immune hemolysis triggered by intravascular red blood cell (RBC) fragmentation resulting from substances in the small blood vessels that produce schistocytes in the peripheral circulation is known as microangiopathic hemolytic anemia. Abnormalities of the microvasculature, including small capillaries and arterioles, are typically implicated. Poor deformability of RBCs can lead to various consequences, including entrapment, phagocytosis, antibody-mediated removal (via phagocytosis or direct complement activation), fragmentation (due to microthrombi or severe mechanical stress), oxidation, or spontaneous cellular death [2]. The enzyme A Disintegrin and Metalloprotease with Thrombospondin motif type 1, member 13 (ADAMTS13) decreases substantially in TTP. This enzyme is necessary for breaking down the von Willebrand factor (vWF), an essential protein in blood coagulation. Abnormally large von Willebrand factor (vWF) multimers accumulate in the circulation and on the surface of endothelial cells when ADAMTS13 is absent. Platelets are drawn to these large multimers, causing platelet-rich clots to form in the smaller blood vessels. These clots can cause obstructions in various organs, leading to ischemia [3]. Though practically every organ can be influenced, the illness most commonly causes acute kidney injury (91%), neurological events (36%–47.4%), stroke or transient ischemic attack (39.5%), altered mental status (15.8%), and seizures (15.8%), as reported by Ping Du *et al*. [4]. Typically, TTP affects 1.5 to 6 cases per million adults annually, making it a relatively uncommon illness. With 10% involving children, adults account for 90% of instances. Women are more prone than men to experience TTP, with a two- to three-fold higher incidence [5]. There are two subtypes of TTP: acquired TTP (aTTP) and congenital TTP (cTTP). Approximately 90%–95% of all TTP cases are immune-mediated, known as acquired TTP [1]. The etiology of aTTP involves the production of autoantibodies against ADAMTS13, which can be either inhibitory or non-inhibitory [6]. aTTP often occurs following an acute episode of infection or inflammation, which can trigger the loss of immunological tolerance and the production of autoantibodies [7]. While Upshaw–Schulman syndrome, also known as cTTP, is an exceptional genetic condition brought on by mutations in the ADAMTS13 gene that result in either diminished or nonexistent plasma von Willebrand factor (VWF) [8]. The five symptoms that were once used to define TTP: fever, thrombocytopenia, microangiopathic hemolytic anemia, neurological symptoms, and renal insufficiency, seem to be no longer used because multiple cohort studies have shown that no more than 10 percent of patients with acute TTP had these symptoms. The nearly invariable symptoms of TTP include severe thrombocytopenia (usually <30 × 10^9^/L) and microangiopathic hemolytic anemia, which can be recognized by schistocytes on the blood smear and sometimes accompanied by related symptoms (such as weakness, dyspnea, and skin and mucosal bleeding) [9]. Anemia, thrombocytopenia, and evidence of active hemolysis are required in laboratory investigations for patients with TTP. Increased reticulocyte count, schistocytes, elevated unconjugated bilirubin, elevated lactate dehydrogenase (LDH), and decreased haptoglobin are the laboratory test results that support hemolysis [10].

In this report, we present a case of aTTP diagnosed in a patient from Jeddah, Saudi Arabia. The case is notable for its atypical clinical course, characterized by the absence of identifiable triggers and a lack of renal involvement, leading to classification as immune-mediated TTP (iTTP). The patient did not exhibit several classical manifestations of TTP, such as renal impairment or fever. Despite receiving appropriate standard-of-care treatment, the patient developed a pulmonary embolism and tested positive for antinuclear antibodies (ANA).

## CASE PRESENTATION

The patient is a 43-year-old Saudi man with a known history of pre-diabetes mellitus, managed with metformin, who initially presented with complaints of epigastric pain. According to limited documentation from the referring hospital, initial investigations revealed thrombocytopenia, which was managed as immune thrombocytopenia with high-dose dexamethasone. However, his condition continued to worsen, and he also developed rashes over the extremities and hematuria, with no history of joint pain, for which he was hospitalized in the other center. Further evaluation revealed severe thrombocytopenia, with a platelet count of 12 × 10^9^/L. Given the lack of response to two platelet transfusions and pulse corticosteroid therapy, a diagnosis of TTP was considered. The patient underwent two sessions of plasmapheresis before being transferred to our intensive care unit (ICU) for further management and care. On admission, the patient was conscious, oriented, and afebrile, with stable vital signs. The abdomen was soft and lax, with epigastric tenderness. Examination of other systems was unremarkable. Investigation reports are shown in Tables 1,2 and 3.

**Table 1:** Laboratory investigations from the day of admission

Complete blood count	Result	Renal function test	Result	Liver function test	Result
White blood cells (WBC)	34x10^9^/L	Sodium	136 mmol/L	**Bilirubin, Total**	**0.113 mmol/L**
Red blood cells (RBC)	**2.52x10^9^/L**	Potassium	3.6 mmol/L	**Bilirubin, Direct**	**0.037 mmol/L**
Hemoglobin (Hb)	**7.9 g/dL**	Glomerular Filtration Rate (GFR) (per 1.73m^2^)	83 ml/min	Serum Albumin	3.6 g/dL
Hematocrit (HCT)	22.3%	Serum Creatinine	0.063 mmol/L	**Serum Total Protein**	**5.2 g/dL**
Mean corpuscular volume (MCV)	88.5 pg	**Serum Blood Urea Nitrogen**	**1.56 mmol/L**	Aspartate Aminotransferase	89 U/L
Platelet Count	**8x10^9^/L**	**Urinalysis**		Alanine Aminotransferase	44 U/L
Reticulocyte count (0.6-2.71%)	**11.49%**	**Red Blood Cells (RBC) (0-2 HPF)**	**10.4**	Alkaline Phosphatase	81 U/L
Haptoglobin (0.3-2 g/L)	**<0.08 g/L**	White Blood Cells (WBC) (0-5 HPF)	1.2	**Lactate Dehydrogenase (135-225 U/L)**	**2107**
Coagulation profile, Serum fibrinogen	Normal				
Peripheral smear	Schistocytes 5%, increased number of reticulocytes				

**Table 2:** Further laboratory investigations

Test	Result
**Antinuclear antibody (ANA)**	**Positive**
Coomb’s Test	Negative
Malaria smear	Negative
HIV serology	Negative
Hepatitis B serology (including Hepatitis B Core)	Negative
Hepatitis C serology	Negative
Troponin	Normal

**Table 3:** Radiological investigations

Test	Result
Ultrasound of the abdomen and pelvis	**Coarse hepatomegaly and diffuse liver disease** **Mild splenomegaly** **Mild cystitis and mild enlarged prostate**
**Echocardiogram**	Normal

Based on the investigations, other differentials were ruled out, and a diagnosis of TTP was confirmed while awaiting ADAMTS13 assay results. A hematologist was brought on board, who suggested that the positive ANA titer could be attributed to the development of an autoimmune component. Consequently, the patient was initiated on intravenous methylprednisolone at a dose of 1 g once daily. The pain was managed using dexmedetomidine and fentanyl, and he was put on a sliding insulin scale for glycemic control. He underwent two more rounds of plasmapheresis. Blood cultures obtained from the central venous access site grew *Staphylococcus epidermidis*, for which the patient was treated with vancomycin 1 g IV, administered as intermittent infusions twice daily for 3 days.

**Figure 1:**
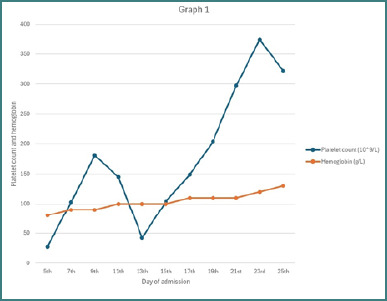
Serial trends in platelet count and hemoglobin levels during hospitalization

**Figure 2:**
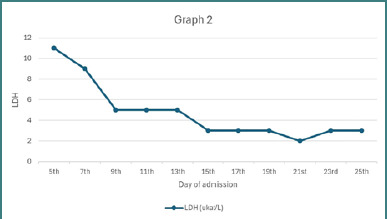
Lactate dehydrogenase (LDH) trend reflecting hemolytic activity over time

**Figure 3:**
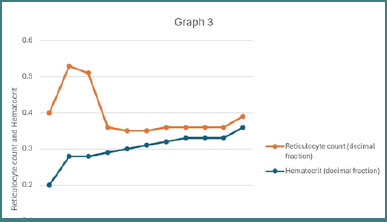
Changes in reticulocyte count and hematocrit during clinical course

Two days after admission, the patient developed complaints of blurry vision, which was followed by bouts of confusion that resolved within a day. Ophthalmological and neurological examinations, including CT, MRA, MRV, and MRI, revealed no abnormalities. It was concluded that this was a self-limited episode of neurological manifestation following the expected symptoms of TTP. The patient was started on prophylactic enoxaparin 40 mg subcutaneously once daily. Over the course of nine sessions of plasmapheresis and transfusion of one unit of packed red blood cells (PRBCs), the patient demonstrated continuous clinical improvement. However, two days after plasmapheresis was stopped, the patient developed a headache accompanied by a declining platelet count and a concurrent rise in serum lactate dehydrogenase (LDH) levels, as illustrated in Figures 1–3. Potential alternative causes, including infection and malignancy, were excluded (Table 4).

**Table 4:** Laboratory and imaging studies excluding other pathologies contributing to disease progression

Test	Result
Carcinoembryonic Antigen (CEA)	Normal
Prostate Specific Antigen (PSA)	Normal
**Computed Tomography (CT) Scan Chest and Abdomen**	**Pulmonary embolism in the right descending pulmonary artery** Right lower lobe non-specific nodules
Quantiferon Tuberculosis Gold Interferon Gamma Release	Negative
Heparin Induced Thrombocytopenia (HIT) Assay	Negative

**Figure 4:**
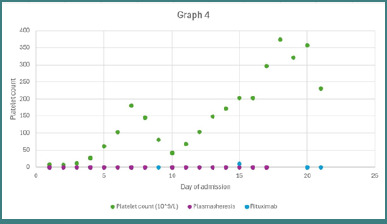
The course of disease progression with respect to medical intervention

ADAMTS13 assay reported <10% ADAMTS13 activity, and ADAMTS13 antibody levels were 88 U/mL, thus confirming the diagnosis of acquired iTTP. He was started on rituximab 375mg/m^2^ weekly for 4 weeks, suspecting early relapse. A CT scan of the chest, performed to exclude underlying malignancy, instead identified a pulmonary embolism in the right descending pulmonary artery. Consequently, enoxaparin was discontinued, and anticoagulation was switched to fondaparinux 7.5 mg subcutaneously once daily. The absence of deep vein thrombosis was ensured using bilateral lower limb venous Doppler. He further underwent sessions of plasmapheresis. This comprehensive treatment approach led to clinical stabilization and progressive improvement in platelet counts (Figure 4). He was discharged following a total of 16 plasmapheresis sessions and three doses of rituximab. The fourth rituximab dose was administered on October 2, 2024, during outpatient follow-up.

## PATIENT PERSPECTIVE

When asked about his experience, the patient reflected on the significant physical and psychological toll exerted by both the disease and its treatment. He described the hospitalization period as highly distressing, with considerable anxiety and fatigue associated with the repeated procedures. However, during follow-up, he reported substantial improvement in his overall condition. He was free of symptoms and expressed a sense of relief and well-being compared to his state at the time of admission.

## DISCUSSION

In the case of acute TTP, it is imperative to receive immediate medical attention, such as immunosuppressive therapy and plasma exchange, to prevent potentially fatal consequences, including organ failure. Although the use of plasmapheresis has significantly increased survival rates, the death rate for patients receiving standard care still ranges from 7% to 22%. Delayed diagnosis and initiation of therapy remain key contributors to both increased mortality and the risk of relapse. Acquired TTP is more prevalent than the congenital variant and is caused by autoantibodies targeting ADAMTS13. Several clinical conditions have been associated with the generation of these autoantibodies, including bacterial infections, autoimmune diseases such as antiphospholipid syndrome and systemic lupus erythematosus, pregnancy, HIV infection, pancreatitis, malignancies, and organ transplantation. Drugs such as ticlopidine, mitomycin C, cyclosporine, quinine, clopidogrel, and estrogen-containing birth control are also associated with TTP. Some of the most widely cited surgeries connected with TTP are vascular and orthopedic procedures, which tend to have high rates of hemolysis and shear stress that may predispose individuals to developing TTP [5,11,12].

The patient initially presented with epigastric pain, a non-specific symptom that can potentially divert clinical suspicion away from TTP. Further analysis revealed thrombocytopenia, which was treated as immune thrombocytopenia with high-dose dexamethasone. However, his condition continued to worsen, and he developed rashes over his extremities and hematuria. The patient had severe thrombocytopenia (platelet count 12x10^9^/L) and received two platelet transfusions and pulse steroids with no improvement. On the day of admission to our hospital, laboratory studies showed platelet count 8x10^9^/L, schistocytes 5%, increased number of reticulocytes, hemoglobin 7.9 g/dL, and LDH at 2107 U/L, which can point the diagnosis to one of these differentials: thrombotic thrombocytopenic purpura, disseminated intravascular coagulation (DIC), hemolytic uremic syndrome (HUS). Since coagulation is typically intact in TTP, the presence of normal prothrombin time (PT), partial thromboplastin time (PTT), and fibrin degradation products (FDP) may be used in certain situations to differentiate TTP from DIC. While HUS shares overlapping clinical features with TTP, it is more frequently encountered in the pediatric population and is typically characterized by more severe renal impairment and less pronounced thrombocytopenia. Although there are exceptions, these distinctions aid in clinical differentiation. TTP can be verified by additional laboratory testing that measures anti-ADAMTS13 antibodies and ADAMTS13 activity [11]. In our patient, a provisional diagnosis of TTP was made based on clinical and laboratory findings, and plasmapheresis was initiated empirically after excluding alternative etiologies. This diagnosis was subsequently confirmed by ADAMTS13 testing, which revealed severely reduced enzymatic activity and elevated inhibitor titers, consistent with acquired immune-mediated TTP.

Acquired TTP has been mostly associated with the risk factors mentioned earlier. Surprisingly, our patient had no family history of autoimmune illnesses, a notable medical history, and none of the risk factors associated with TTP. Based on clinical, laboratory, and immunologic findings, the final diagnosis was primary immune-mediated TTP. In addition, the patient tested positive for ANA, a finding observed in approximately 27.8% of patients with primary immune-mediated TTP, although it does not necessarily indicate a currently coexisting connective tissue disease. Longitudinal studies have shown that individuals recovering from TTP are at increased risk, up to 12 years post-episode, of developing autoimmune diseases, particularly systemic lupus erythematosus (SLE) and Sjögren’s syndrome. This confirms the need to treat TTP as a chronic autoimmune disease, necessitating a careful, extended period of follow-up [13,14]. This could aid in clinical decision-making and shorten the time to diagnosis and appropriate therapy, emphasizing the need for more research to completely understand the mechanisms by which TTP occurs [11].

It is challenging to diagnose a case of TTP in the absence of the classic pentad presentation. Furthermore, acute kidney injury is one of the most common short-term illness consequences for iTTP, accounting for 91% of cases [4]. Our case was unique in that significant renal involvement was absent, and we relied on excluding other differentials. However, the use of a PLASMIC score, which is based on a cohort study that identified many variables among patients with TTP, is one of the current guidelines for early diagnosis. This score assigns one point for each of the following criteria: hemolysis (reticulocyte count >2.5%, total bilirubin >2.0 mg/dL, or decreased haptoglobin), mean corpuscular volume (MCV) <90 fL, international normalized ratio (INR) <1.5, serum creatinine <2.0 mg/dL, absence of active cancer, and no history of solid organ or stem cell transplantation. A score of ≤4 indicates low probability, while a score ≥6 suggests high probability of TTP. In our case, the patient scored 7 on the PLASMIC scale, placing him in the high-risk category with an estimated 72% likelihood of having an ADAMTS13 activity level <15% [11,15]. A second score called the French score, is calculated by assigning one point for each of the following: platelet count ≤ 30 × 10^9^/L, creatinine level ≤ 2.26 mg/dL, and positive ANA. Our patient was categorized as high-risk, accounting for two points [16].

A correlation exists between increased mortality and delays of more than 24 hours in initiating plasma exchange after presentation, highlighting the critical importance of prompt diagnosis and treatment [11]. Until recently, immunosuppressive medications such as corticosteroids and daily therapeutic plasma exchange (TPE) were the mainstay treatments for acute TTP. PEX is frequently used with corticosteroid therapy to inhibit the generation of autoantibodies against ADAMTS13. Although most patients respond well to PEX, recurrence rates can reach 50% to 60%. This case demonstrates that rituximab can enhance the therapeutic effectiveness of PEX and shield TTP patients from disease recurrence [15]. Recently, caplacizumab-based anti-vWF medication was approved as part of first-line therapy for acute TTP in combination with corticosteroids and plasma exchange (PEX). Current guidelines suggest using it for acute TTP because the first 10 days following diagnosis are often lethal for thrombosis, and immunosuppressive medication takes time to show results [17]. In the United Kingdom, a relapse rate of 3.5% was reported among patients receiving caplacizumab in combination with other standard therapies, as per physician decision. Additionally, 1.1% of iTTP patients in France were treated with a triplet regimen of PEX, immunosuppression with corticosteroids, rituximab, and caplacizumab [4]. In our case, the patient was promptly initiated on PEX and corticosteroids upon clinical suspicion of TTP. He was planned to receive caplacizumab, but due to an insurance issue, he did not receive it. Following appropriate management, the patient was monitored to assess the response to treatment.

Patients who require further medication after failing to respond to PEX and corticosteroids have a reported incidence ranging from 10% to 42%. According to the 2012 American Society of Apheresis Consensus Conference on TTP, treatment response is defined by a platelet count >150,000/μL on two consecutive days, normalization or near-normalization of lactate dehydrogenase LDH levels, and stabilization or improvement of neurological symptoms [18]. In contrast, the British Committee for Standards in Haematology defines treatment failure as the inability to achieve a platelet count >150 × 10^9^/L and/or an LDH level <1.5 times the upper limit of normal (1.5N), along with the appearance of new or worsening ischemic organ symptoms after five therapeutic plasma exchange (TPE) sessions. Recurrence of any TTP symptom following a period of clinical remission, up to 30 days following the conclusion of medication, is referred to as an exacerbation. Remission is characterized by a clinical response that lasts more than 30 days following treatment discontinuation. Any symptom of TTP that reappears after remission—a decline in ADAMTS13 activity or clinical or biochemical indicators of thrombotic microangiopathy—is referred to as a relapse. Refractory TTP is defined as the failure to achieve a platelet count >150 × 10^9^/L after seven TPE sessions [19]. In our case, the patient’s platelet count reached 181 × 10^9^/L by the fifth day of receiving PEX and corticosteroid therapy, thereby fulfilling the criteria for treatment response. As a result, PEX was temporarily discontinued. However, following a two-day cessation of PEX, the patient exhibited a decline in platelet count, which dropped to 80 × 10^9^/L on the third day after treatment was stopped, necessitating the resumption of plasma exchange. This clinical course does not fit neatly within existing definitions of refractory disease, exacerbation, or relapse, as the patient initially responded appropriately to therapy, and deterioration occurred shortly after the temporary discontinuation of treatment. In contrast, his LDH on the day of admission was 2107 U/L and started to trend down after receiving treatment. On the day of deterioration, it normalized, reaching 175 U/L.

During follow-up, the patient was closely monitored to exclude potential complications and alternative causes of clinical deterioration, including infection and malignancy. While malignancy was ruled out, a CT scan of the chest and abdomen incidentally revealed a pulmonary embolism (PE) in the right descending pulmonary artery without any corresponding symptoms. This can be correlated with a recent report showing an association between venous thromboembolism (VTE) and plasma exchange treatment for TTP using solvent detergent (SD) plasma, as VTE is not a feature of TTP. Specifically, class I graduated elastic compression stockings should be initiated at diagnosis, and prophylactic low-molecular-weight heparin is advised once the platelet count exceeds 50 × 10^9^/L [20]. Our case study highlights the need for further research and standardization of definitions to enhance understanding of disease progression and treatment.

## CONCLUSION

Thrombotic thrombocytopenic purpura is a life-threatening illness caused by an abnormality in ADAMTS13 enzyme activity, leading to irregular blood clotting and organ damage. Timely identification and treatment are essential to avoid tragic results; these treatments usually involve immunosuppressive therapy and plasma exchange. The acquired form of TTP is more common and may be triggered by medications, autoimmune diseases, infections, or other underlying conditions. Although corticosteroids and PEX therapies increase survival, recurrence and refractory cases continue to be challenging to treat. New treatments, like caplacizumab, give hope for improved results. However, as illustrated in this case, diagnostic uncertainty, atypical presentations, and variable treatment responses can complicate clinical management. To cure and better understand TTP and manage its side effects, including venous thromboembolism associated with PEX therapy, further research is required, as well as the development of improved evaluation tools, such as the PLASMIC score.
